# Development of a Computerized Adaptive Testing for Internet Addiction

**DOI:** 10.3389/fpsyg.2019.01010

**Published:** 2019-05-07

**Authors:** Yong Zhang, Daxun Wang, Xuliang Gao, Yan Cai, Dongbo Tu

**Affiliations:** School of Psychology, Jiangxi Normal University, Nanchang, China

**Keywords:** internet addiction, computer adaptive testing, item response theory, questionnaire, CAT-IA

## Abstract

Internet addiction disorder has become one of the most popular forms of addiction in psychological and behavioral areas, and measuring it is growing increasingly important in practice. This study aimed to develop a computerized adaptive testing to measure and assess internet addiction (CAT-IA) efficiently. Four standardized scales were used to build the original item bank. A total of 59 polytomously scored items were finally chosen after excluding 42 items for failing the psychometric evaluation. For the final 59-item bank of CAT-IA, two simulation studies were conducted to investigate the psychometric properties, efficiency, reliability, concurrent validity, and predictive validity of CAT-IA under different stopping rules. The results showed that (1) the final 59 items met IRT assumptions, had high discrimination, showed good item-model fit, and were without DIF; and (2) the CAT-IA not only had high measurement accuracy in psychometric properties but also sufficient efficiency, reliability, concurrent validity, and predictive validity. The impact and limitations of CAT-IA were discussed, and several suggestions for future research were provided.

## Introduction

Internet addiction (IA) disorder is now recognized as one of the most popular forms of addiction in psychological and behavioral areas. According to a report released by the International Telecommunication Union (2016), with the rapid development of advanced mobile networks, the number of users over the last 3 years has climbed to nearly four billion people, which is equivalent to 47% of the global population. Although the internet brings many benefits, excessive access to the network can lead to internet addiction (IA). A recent meta-analysis reported that the global prevalence of IA is 30.1% among university students pursuing a professional degree (Zhang et al., 2018). In Asia, the prevalence of IA ranged from 6.2% in Japanese adolescents to 21% in Filipino adolescents (Mak et al., 2014b). IA is associated with sleep disturbance (Zhang et al., 2017), poor quality of life (Tran et al., 2017a), and other psychiatric illnesses (Ho et al., 2014). Therefore, the assessment and prevention of IA are particularly important in practice. IA symptoms have been evaluated primarily by questionnaires that have been developed based on classical test theory. The commonly used questionnaires include the Internet Addiction Test (IAT; Young, 1998), Generalized Problematic Internet Use Scale (GPIUS; Caplan, 2002), Gaming Addiction Scale (GAS; Lemmens et al., 2009), and Revised Chen Internet Addiction Scale (CIAS-R; Mak et al., 2014a). The current questionnaires classify IA symptoms into loss of control or of time management (Tran et al., 2017b), craving and social problems (Lai et al., 2013). Although these questionnaires are frequently used in practice, they have certain weaknesses. One of the most notable drawbacks is that participants must finish all of the questionnaire items. However, many items may be “off target” for different test takers (Fliege et al., 2005). For participants with high ability levels, easy items have less contribution to measuring their actual ability level, and as such, these items may be redundant or unnecessary. Meanwhile, for participants with low ability levels, the requirement of responding to the difficult items results in the difficulty to measure their actual ability level. Therefore, it is essential to have a more effective method to evaluate IA.

One way to deal with the above issues is through computerized adaptive testing (CAT), which is a new kind of test that uses item response theory (IRT) to establish an item bank, and then automatically selects items according to the current theta of each participant, and finally estimates the ability of each test taker (Almond and Mislevy, 1999). In CAT, the test-taker continues to take test items until his/her estimated θ reaches a predefined level of precision, as indicated by its standard error. Compared with a linear test, CAT cannot only present items, input answers, and automatically score through the computer but also automatically select the most appropriate items for each responder according to the different answers to items, and then finally reach the most appropriate estimation of ability.

Many studies have shown that a CAT program has several advantages over paper-and-pencil questionnaires. Flens et al. (2016) revealed that compared with paper-and-pencil questionnaires, the number of used items based on CAT procedures decreases by 26–44%. Linacre (2000) pointed out that CAT programs can improve validation, reduce individuals’ burden, and have more excellent measurement precision. In addition, with the selection of items based on a respondent’s current theta, the floor and ceiling effects can be decreased in CAT procedures (Revicki and Cella, 1997). Further, the development of CAT procedures improves clinical assessment. However, CAT also has a number of disadvantages: high costs of research and development, complex technical requirements, and the need for timely maintenance of the item bank to prevent items from leaking in advance (Tan et al., 2018). Nonetheless, the virtues of a CAT program importantly overweigh the defects.

Initially, the development and applications of CAT programs mainly occurred in intelligence and ability testing (e.g., Tinsley, 1972; Ireland, 1977; Young, 1990). In recent years, many researchers have paid attention to the field of mental health. For example, Flens et al. (2017) used the IRT model to assess the Dutch-Flemish version of depression. Smits et al. (2011) established and evaluated CAT procedures for depression based on the Epidemiologic Studies-Depression scale. Walter et al. (2007) developed a German version of Anxiety CAT within IRT. However, to the best of our knowledge, the use of CAT to IA, a common disorder, has not been applied.

This study aimed to develop CAT to assess IA (CAT-IA) without loss of measurement precision. More specifically, this work addressed the following. First, a calibrated item bank with high psychometric qualities was developed. Second, in different stopping rules, we evaluated the psychometric properties, efficiency, reliability, and validity of CAT-IA via two CAT simulation studies. Third, we sought to extend the applications of CAT in the field of mental health and introduce IRT and CAT to readers who want to understand and apply adaptive testing.

## Materials and Methods

### Participants

The total sample consisted of 1,368 participants. All of the participants were surveyed at different schools in China from June to September 2017. Table 1 reveals the characteristics of the participants. The sample included 687 females (50.2%) and 681 men (49.8%). Their average age was 18.72 years (*SD* = 2.19, ranged from 12 to 28 years). The participants came from two regions: rural (58.9%) and urban (41.1%).

**Table 1:** The characteristics of participants (*n* = 1,368).

Characteristics	% or years
**Gender**	
Female	50.2
Male	49.8
**Age**	
Mean	18.72
SD	2.19
Range	12–28
**Region**	
Rural	58.9
Urban	41.1


This study was conducted at the Research Center of Mental Health, Jiangxi Normal University, following the recommendations of psychometrics studies on mental health. It was approved by the Research Center of Mental Health, Jiangxi Normal University and the Ethics Committee of the Department of Psychology at Jiangxi Normal University. Written informed consent was obtained from all of the participants in accordance with the Declaration of Helsinki. Parental consent was also obtained for all participants under the age of 16 years.

### Measures and the Initial Item Pool

The initial item pool of CAT-IA consisted of 101 items (see Table 2). These items were selected from four standardized scales: IAT (Young, 1998), GPIUS (Caplan, 2002), GAS (Lemmens et al., 2009), and Chinese Internet Addiction Test (CIAT; Huang et al., 2007). All of them used five-point Likert-type item scores (never, rarely, sometimes, often, always; scored with 1, 2, 3, 4, and 5, respectively). A higher cumulative sum in all of the items represented more severe symptoms of IA. Based on previous studies, 101 items from the four selected standardized scales could be classified into seven domains (Young, 1998; Caplan, 2002; Huang et al., 2007; Lemmens et al., 2009): salience, tolerance, mood modification, relapse, withdrawal, negative outcomes, and benefits (i.e., compared with offline, individuals are more likely to participate in social behavior online and surfing the internet can reduce negative emotions).

**Table 2:** Items from four scales.

Scale	Number of items	Items
IAT	20	IAT-1, 2, 3, 4, 5, 6, 7, 8, 9, 10, 11, 12, 13, 14, 15, 16, 17, 18, 19, and IAT-20
GPIUS	29	GPIUS-21, 22, 23, 24, 25, 26, 27, 28, 29, 30, 31, 32, 33, 34, 35, 36, 37, 38, 39, 40, 41, 42, 43, 44, 45, 46, 47, 48, and GPIUS-49
GAS	21	GAS-50, 51, 52, 53, 54, 55, 56, 57, 58, 59, 60, 61, 62, 63, 64, 65, 66, 67, 68, 69, and GAS-70
CIAT	31	CIAT-71, 72, 73, 74, 75, 76, 77, 78, 79, 80, 81, 82, 83, 84, 85, 86, 87, 88, 89, 90, 91, 92, 93, 94, 95, 96, 97, 98, 99, 100, and CIAT-101


*IAT, Internet Addiction Test; GPIUS, Generalized Problematic Internet Use Scale; GAS, Gaming Addiction Scale; CIAT, Chinese Internet Addiction Test.*

### Item Bank Construction of CAT-IA

To obtain a high-quality item bank, psychometric evaluations were performed on the individuals’ actual data as follows.

**Step 1:** Test the unidimensional assumption of the item pool.

Unidimensionality means that the test measures only one main latent trait; that is, responses on each item are affected by one main latent trait of the participants (Embretson and Reise, 2013). Both exploratory factor analysis (EFA) and confirmatory factor analysis (CFA) were used to assess the unidimensional assumption. In EFA, the unidimensional assumption is deemed sufficient when the first factor explains at least 20% of the variance (Reckase, 1979), and the ratio of the explained variance in the first and second factor is greater than 4 (Reeve et al., 2007). The CFA of a single-factor was used to assess the unidimensional assumption. We adopted two indicators: factor loading and root mean square error of approximation (RMSEA) estimated by the weighted least square means and variance adjusted method using Mplus7.0 (Muthén and Muthén, 2012). According to the rule of thumb of Browne and Cudeck (1993), the model has a close fit, is fair or acceptable, mediocre, or poor if the RMSEA value is below 0.05, between 0.06 and 0.08, between 0.09 and 0.10, or above 0.10, respectively. We excluded items with factor loadings smaller than 0.4 because factor loadings below 0.4 could easily be over-interpreted (Nunnally, 1978).

**Step 2:** Select the appropriate IRT model according to the test-level model-fit indices.

Selecting the appropriate model is one of the most important procedures to make valid inferences. In this study, four commonly used polytomous IRT models were considered: Graded Response Model (GRM; Samejima, 1969), Generalized Partial Credit Model (GPCM; Muraki, 1992), Graded Ratings Scale Model (GRSM; Andrich, 1978), and Nominal Response Model (NRM; Bock, 1972). The test-level model-fit indices were used to compare and select IRT models, which included Akaike’s information criterion (AIC; Akaike, 1974), Bayesian information criterion (BIC; Schwarz, 1978), and -2Log-Likelihood (-2LL; Spiegelhalter et al., 1998). The smaller values of these indices showed the better model fit; therefore, the model with the smallest test-fit indices was selected for further analysis. Model selection analysis was done in R package mirt (Version 1.10; Chalmers, 2012).

**Step 3:** Assess the local independence of the remaining items in the item pool.

Local independence includes two aspects: one is that the response of the same participants (or similar-level participants) to any one item will not be affected by any other items on the same test; and the other is that the responses of different participants (or different-level participants) on the same item do not affect each other (Embretson and Reise, 2013). Currently, the Q3 statistic (Yen, 1993) is commonly used to verify the dependent relationship between items. We calculated the Q3 values of any two items from the item pool under the selected IRT model in Step 2, via R package mirt (Version 1.10; Chalmers, 2012). As suggested by Cohen (2013), Q3 values below 0.36 represented local independence. Hence, one item with Q3 > 0.36 in item pairs was removed.

**Step 4:** Assess the monotonicity of the remaining items in the item pool.

Monotonicity, meaning that a person with higher latent trait levels raises the possibility of higher scores for an item, was assessed by scalability coefficients for the item pool and individual items via R package Mokken (Version 2.7.7; van der Ark, 2007). According to Mokken (1971), a scale or item has high quality if the scalability coefficient is above 0.3. Items with scalability coefficients below 0.30 were thus eliminated until all of the scalability coefficients exceeded 0.3.

**Step 5:** Analyze the psychometric characteristics of the remaining items in item pool.

After items were excluded in the above four steps, psychometric characteristics (i.e., item-fit, differential item functioning [DIF], and discrimination) were evaluated for the remaining items. First, the S-X^2^ statistic (Orlando and Thissen, 2003) was used to exam item fit using R package mirt (Version 1.10; Chalmers, 2012). Second, ordinal logistic regression, a nimbler method in detecting DIF, was used to test DIF for gender (male and female), age (under 18 years, and 18 and above), and region groups (rural and urban), respectively, via R package lordif (Version 0.2-2; Choi et al., 2011). DIF was assessed by means of change in McFadden’s *R*^2^ between different groups; items with *R*^2^ change greater than 0.02 indicated DIF (Choi et al., 2011). The item parameters, namely, the discrimination (a) and difficulty parameters (b), were estimated under the selected model.

**Step 6:** Choose high-quality items to develop the final item bank of CAT-IA.

According to the psychometric characteristics in Step 5, poor model-fit (*p* < 0.01), DIF, and low discrimination items (*a* < 1.00) were all excluded. This procedure was repeated until no item was excluded.

### CAT Simulation

To evaluate the psychometric properties, efficiency, reliability, concurrent validity, and predictive validity of CAT-IA, two CAT simulation studies were carried out. A CAT study is generally composed of six parts: the item bank, item response models, selection methods of initial items, evaluation methods of latent trait, item selection methods, and the stopping rules (Weiss and Kingsbury, 1984). First, the 59-item bank of CAT-IA was established, and the item parameters were estimated under the selected IRT model. Second, an item from the 59-item bank was randomly selected as the initial item to control the exposure rate. Ability estimation methods mainly include maximum likelihood estimation (MLE), weighted likelihood estimation (WLE), maximum a posteriori estimation (MAP), and expected a posterior estimation (EAP) in CAT procedures (e.g., Chen et al., 1998; Wang and Vispoel, 1998; Gorin et al., 2005). The MAP, MLE, and EAP methods regard the maximum point of the likelihood function (or posterior distribution) as the estimated ability value, which may result in multiple extreme points at the beginning of tests (Magis and Raîche, 2010). However, the mean value of the whole posterior distribution is adopted in EAP algorithm. Thus, the information provided by the entire posterior distribution can be effectively utilized, and the stability of the EAP algorithm is higher than that of the other three methods. The EAP method uses the mean value of the entire posterior distribution; therefore, it need not be iterated, and the calculation process is simpler. Compared with the MLE and WLE methods, the EAP method has a larger bias and belongs to biased estimation (Wang et al., 1999). Compared with the EAP method, the main advantage of MAP is that it requires fewer items in the variable-length test, which means that the test is more efficient (Wang and Vispoel, 1998). However, the virtues of the EAP algorithm importantly overweigh its drawbacks. The simplicity and stability of the EAP method makes it an optimal method for CAT simulations (e.g., Warm, 1989; Chen et al., 1998; Bulut and Kan, 2012). Further, maximum information criterion (MIC; Lord, 1980) is the most widely used item selection strategy in CAT programs because of its relatively simple implementation method. The purpose of this strategy is to improve the accuracy of measurement (Brunel and Nadal, 1998), but it can easily lead to uneven exposure of items in the item bank and reduced security of the test (Barrada et al., 2008). Different from the exam, a Likert-type scale without correct answers requires participants to respond in the usual way, which greatly reduces the test security problem. Therefore, MIC was selected as the item selection method in the CAT-IA simulation study. Finally, several stopping rules with different SEs were performed, including None (i.e., the entire item bank was used), SE ≤ 0.2, SE ≤ 0.3, SE ≤ 0.4, and SE ≤ 0.5, respectively.

#### Simulation Study 1: Psychometric Properties of CAT-IA

When a CAT-IA program is established, its psychometric properties should be evaluated, especially in terms of measurement accuracy. The results of CAT-IA may result in high-risk outcomes that are similar to the entrance exam. Therefore, the Monte-Carlo (MC) simulation method was used to evaluate the performance of CAT-IA. First, the ability of 1,000 virtual persons were generated randomly from the normal distribution (Mean = 0, *SD* = 1); this sample was regarded as the true ability values. Second, the item parameters of the final 59-item bank and selected IRT model were used to conduct the CAT-IA simulation study. Third, the MC method was used to estimate the ability value of each participant according to the true θ values, selected IRT model and item parameters. These abilities were the estimated values of 1,000 simulated persons. In addition, the CAT-IA performance was evaluated via several statistical indices, including conditional bias (CBIAS), conditional mean absolute error (CMAE), conditional root mean square error (CRMSE), and conditional standard error of estimation (CSEE) across all θ areas (Han, 2018). Simulation study 1 was done in the R package catR (Version 3.12; Magis and Barrada, 2017). These statistical indices for every participant were plotted under different stopping rules using SPSS (Version 23.0; George, 2016).

#### Simulation Study 2: Efficiency, Reliability, and Validity of CAT-IA

##### Efficiency and reliability of CAT-IA

To evaluate the efficiency and reliability of CAT-IA, a simulation based on the actual data was carried out via the R package mirtCAT (Version 0.5; Chalmers, 2015). In simulation study 2, the real responses to items were used instead of virtual responses generated by the MC method; the process of simulation study 2 was the same as that in simulation study 1. For each responder, the SE could be calculated in simulation study 2. Green et al. (1984) pointed out that a unitless reliability index is necessary for a CAT, even if this index is somewhat contrived. The index of marginal reliability was proposed by Green et al. (1984) to evaluate effectively the reliability of a CAT under different stopping rules. Marginal reliability is a relatively convenient way to monitor dynamically the reliability of a CAT, and can also be used to evaluate the stability of a CAT (Green et al., 1984). In general, marginal reliability is a function of standard error of measurement (SEM), as shown in formulas (1) and (2). The bigger the marginal reliability is, the smaller the SEM is. Therefore, marginal reliability is crucial for the assessment of SEM and the reliability of measurement in CAT. Marginal reliability is equal to the mean reliability under each stopping rule for all participants (Wainer et al., 2000b). The formula of marginal reliability is defined as:

(1) $$MR=1-SE^{2}$$

(2) $$SE=\frac{\sum_{i=1}^{N}SE(\theta_{i})}{N}$$

Where *n* is the number of all participants, and *SE*(*θ_i_*) is the standard error of examinee *i* at the finally estimated θ. Some statistics were investigated to examine the efficiency and reliability of CAT-IA, including the mean and standard deviation of the used items, mean SE, marginal reliability, and Pearson’s correlations between the estimated θ with the stopping rule of None and the remaining stopping rules. The number of used items with the reliability for every participant was plotted under different stopping rules using the R package ggplot2 (Version 2.2.1; Wickham, 2011).

##### Concurrent validity and predictive validity of CAT-IA

CAT-IA may take effect when CAT-IA estimation results have a favorable similarity to the results of the existing widely used scales. In other words, a person who is diagnosed with IA in a questionnaire has a higher latent trait in a CAT estimation compared with those without a diagnosis of IA. The similarities were evaluated by concurrent validity and predictive validity of CAT-IA using SPSS (Version 23.0; George, 2016) based on the initial responses that were used to establish the item bank of IA. The concurrent validity was evaluated by the Pearson’s correlations between the estimated θ of CAT-IA and the aggregate scores of each scale. Based on previous studies, only two scales (IAT and GAS) possess the definite diagnostic criteria for IA (Young, 1998; Caplan, 2002; Huang et al., 2007; Lemmens et al., 2009). Individuals whose sum scale scores of IAT exceed 39 are considered as having problematic network usage (Young, 1998). GAS includes seven diagnostic items (Lemmens et al., 2009); individuals with at least four items scoring 4 or 5 are considered to be addicted. The diagnostic results of IAT and GAS were used to compare the estimated results of CAT-IA. Then, the AUC (the area under ROC curve) index was employed to investigate the predictive effect of CAT-IA. According to the rule of Rice and Harris (2005), AUC values below 0.50 represent a small predictive effect; values between 0.51 and 0.70, a moderate predictive effect; and values higher than 0.71, a large predictive effect. In the ROC curve, determination of the critical points adopted the maximal Youden Index (YI = sensitivity + specificity - 1) (Schisterman et al., 2005). The sensitivity indicates the probability of a patient being diagnosed as a patient, and the specificity indicates the probability of a person without the symptoms being diagnosed as a normal person. Sensitivity and specificity are two important reference indicators for the accuracy of critical values, which are both ranged from 0 to 1, with the bigger values representing better predictive validation.

## Results

### Item Bank Construction of CAT-IA

#### Unidimensionality

In EFA, the ratio of variance explained by the first factor was 32.44% higher than the critical standard of 20% (Reckase, 1979), and the ratio of variance explained in the first and second factors was 5.89 higher than the critical standard of 4 (Reeve et al., 2007). In the single-factor CFA, five items were removed (see Table 3) owing to their factor loadings of below 0.4 (Nunnally, 1978). Both the EFA and single-factor CFA were again conducted on the remaining 96 items. The EFA results showed the ratio of variance explained by the first factor was 33.87%, and the ratio of variance explained in the first and second factors was 6.14. Results of the single-factor CFA indicated that the RMSEA value was 0.08, indicating that the single factor model was fair or acceptable; all factor loadings were above 0.4. The above results showed that the remaining 96 items, after deleting five items, basically met the unidimensional hypothesis.

**Table 3:** Reasons for stepwise exclusion of the items.

Excluded reasons	Excluded items
Unidimensionality	IAT-7 and 9, GPIUS-36 and 37 and CIAT-100
Local Independency	IAT-4 and 16; GPIUS-22, 23, 25, 26, 27, 28, 31, 39, 40, 42, and 48; GAS-50, 52, 51, 53, 54, 57, 58, 60, 62, and 63; CIAT-87, 89, and 90
Monotonicity	IAT-1 and 5; GPIUS-21, 30, and 47; CIAT-73
DIF	GAS-61, 64, 67, and 69
*S*-*X*^2^	IAT-2
Discrimination	None


*DIF, different item function; the abbreviated content of each item can be seen in Table 5.*

#### Model Selection

Table 4 documents the model-fit indices, including -2LL, AIC, and BIC, for the four IRT models. Compared with the other three IRT models, the GRSM fitted the worst in that it had the largest -2LL, AIC, and BIC values. Of the remaining three models, the GPCM model had the worst fitting indices. Although the -2LL value of NRM was smaller than that of GRM, the AIC and BIC values of NRM were both higher compared with the GRM. The GRM model overall fitted the remaining 96-item bank best compared with other three. Therefore, GRM was selected for later analysis.

**Table 4:** Model-fit indices.

Model	-2LL	AIC	BIC
GRM	331710.400	332670.500	335217.000
GPCM	333965.400	334925.300	337471.800
GRSM	336329.000	336719.000	337753.500
NRM	331675.600	333211.600	337286.000


*GRM, Graded Response Model; GPCM, Generalized Partial Credit Model; GRSM, Graded Ratings Scale Model; NRM, Nominal Response Model; -2LL, -2Log-Likelihood; AIC, Akaike’s information criterion; BIC, Bayesian information criterion.*

#### Local Independence

A total of 23 pairs of items showed local dependence: their Q3 values were above 0.36 (Cohen, 2013). Thus, 26 items were excluded owing to local dependence, including 2 IAT items, 11 GPIUS items, 10 GAS items, and 3 CIAT items (see Table 3). Then, the Q3 values of the remaining 70-item bank were reassessed, and the results showed all Q3 values were below 0.36.

#### Monotonicity

The scalability coefficient for the remaining 70-item bank was 0.4, which was higher the requirement of 0.3 (Mokken, 1971). However, for the scalability coefficient of the 70 items, there were still six items (see Table 3) with scalability coefficients below 0.3. After excluding these items, we reevaluated the scalability coefficients, and the results showed that the scalability coefficient of the 64-item bank was 0.39, whereas all scalability coefficients of the 64 items were above 0.3.

#### DIF

For the region and age groups, no DIF was found for all 64 items; the means of change in McFadden’s *R*^2^ between different groups were above the minimum requirement of 0.02 (Choi et al., 2011). However, for the gender group, four items (see Table 3), all belonging to GAS, were flagged for DIF. Therefore, we excluded these items and reassessed the DIF of 60 items. The results showed that the means of change in McFadden’s *R*^2^ all were below 0.02 for the region, age, and gender groups.

#### Item-Fit

Only one item (IAT-2) failed to fit the GRM for having a *p*-value of *S*-*X*^2^ that was less than 0.01. After removing this item, the remaining 59 items were reevaluated, and the results showed that the *p*-value of *S-X*^2^ of all the 59 items were above 0.01.

#### Discrimination

Graded Response Model was used again to calibrate the remaining 59 items. The item parameters are listed in Table 5. The discrimination parameters of the 59 items were all above the value of 1 with mean of 1.627 (*SD* = 14.5), which indicated the final item bank was of a high quality.

**Table 5:** Item parameters for 59-item bank with GRM.

Item	Abbreviated	*a*	*b1*	*b2*	*b3*	*b4*	Domain
IAT-3	Excitement	1.587	-0.540	1.054	2.425	3.092	Mood modification
IAT-6	Work suffer	1.369	-1.365	0.098	1.724	3.213	Negative outcomes
IAT-8	Job suffer	1.292	-1.352	0.071	1.723	3.351	Negative outcomes
IAT-10	Block disturbing	1.072	-1.545	0.057	2.049	3.772	Mood modification
IAT-11	Anticipating	1.236	-1.447	0.204	1.656	2.945	Tolerance
IAT-12	Boring and joyless	1.284	-1.344	-0.202	1.356	2.633	Withdrawal
IAT-13	Annoyed	1.473	-0.466	1.175	2.519	3.457	Withdrawal
IAT-14	Lose sleep	1.397	-0.932	0.499	1.846	3.331	Negative outcomes
IAT-15	Preoccupied	1.863	-0.521	0.797	2.14	3.144	Salience
IAT-17	Fail to reduce time	1.474	-1.258	0.027	1.415	2.648	Relapse
IAT-18	Hide online time	1.302	-0.238	1.255	2.694	3.956	Negative outcomes
IAT-19	Prefer online	1.630	-0.190	1.079	2.316	3.149	Salience
IAT-20	Depressed or nervous	1.972	-0.183	1.133	2.298	3.202	Withdrawal
GPIUS-24	Feel better	1.352	-1.352	-0.303	0.838	2.852	Mood modification
GPIUS-29	Treated better	1.324	-0.725	0.600	1.916	3.438	Benefits
GPIUS-32	Feel worthless offline	1.397	0.217	1.532	2.541	4.121	Benefits
GPIUS-33	Missed social event	1.298	-0.274	0.975	2.092	3.320	Negative outcomes
GPIUS-34	Unsuccessful	1.602	-0.829	0.277	1.209	2.686	Relapse
GPIUS-35	Fail to reduce time	1.631	-0.659	0.452	1.495	2.661	Relapse
GPIUS-38	Forget the time	1.101	-1.525	-0.367	0.595	2.595	Tolerance
GPIUS-41	Longer time	1.404	-1.477	-0.382	0.495	2.531	Tolerance
GPIUS-43	Miss	1.657	-0.976	0.078	0.939	2.545	Withdrawal
GPIUS-44	Wonder	1.335	-1.23	-0.074	0.836	2.867	Withdrawal
GPIUS-45	Feel lost	1.856	-0.675	0.358	1.225	2.696	Withdrawal
GPIUS-46	Unable to stop thinking	1.659	-0.578	0.516	1.481	2.785	Tolerance
GPIUS-49	Control	1.247	-0.400	0.858	2.204	3.795	Benefits
GAS-55	Unable to stop playing	1.381	-0.418	0.848	2.147	3.081	Tolerance
GAS-56	Forget about real life	1.534	-0.099	1.211	2.602	3.401	Mood modification
GAS-59	Unable to reduce time	1.490	-0.207	1.163	2.294	3.205	Relapse
GAS-65	Fights with others	1.719	-0.173	0.960	2.179	3.142	Negative outcomes
GAS-66	Neglected others	1.787	-0.365	0.635	1.967	2.995	Negative outcomes
GAS-68	Lose sleep	1.721	-0.32	0.777	1.948	2.795	Negative outcomes
GAS-70	Feel bad	1.195	-1.299	-0.119	1.611	3.131	Negative outcomes
CIAT-71	Neglect household	1.984	-0.722	0.462	1.687	2.714	Negative outcomes
CIAT-72	Excitement	2.294	-0.374	0.794	1.806	2.629	Mood modification
CIAT-74	Complain of others	1.745	-0.449	0.906	2.061	2.981	Negative outcomes
CIAT-75	School or work suffer	1.879	-0.848	0.334	1.544	2.628	Negative outcomes
CIAT-76	Defensive or secretive	1.189	-0.845	0.767	2.322	3.384	Negative outcomes
CIAT-77	Disturbing	1.631	-1.006	0.152	1.614	2.733	Mood modification
CIAT-78	Anticipating	1.975	-0.742	0.445	1.695	2.513	Tolerance
CIAT-79	Annoyed act	1.831	-0.176	1.157	2.132	2.994	Withdrawal
CIAT-80	Lose sleep	1.456	-0.498	0.739	1.975	2.876	Negative outcomes
CIAT-81	Preoccupied	2.639	-0.404	0.728	1.782	2.407	Salience
CIAT-82	“Just a few minutes”	2.053	-0.866	0.206	1.354	2.421	Relapse
CIAT-83	Hide online time	1.873	-0.207	1.095	2.122	3.108	Negative outcomes
CIAT-84	Spend more time	2.409	-0.375	0.556	1.429	2.242	Tolerance
CIAT-85	Important	2.077	-0.343	0.631	1.63	2.521	Salience
CIAT-86	More attractive	2.093	-0.337	0.744	1.904	2.795	Benefits
CIAT-88	Exciting information	1.382	-1.450	-0.112	1.581	2.845	Benefits
CIAT-91	Reduce the stress	1.443	-1.298	-0.006	1.659	2.918	Benefits
CIAT-92	Times goes faster	1.189	-1.968	-0.854	0.511	2.143	Tolerance
CIAT-93	Stay online	2.192	-0.787	0.404	1.380	2.349	Tolerance
CIAT-94	Want to stay online	2.233	-0.825	0.421	1.605	2.350	Withdrawal
CIAT-95	Disturbed	1.219	-1.951	-0.632	0.800	2.250	Withdrawal
CIAT-96	Distraught	1.894	-0.713	0.511	1.622	2.621	Withdrawal
CIAT-97	Failed to reduce time	2.103	-0.698	0.493	1.575	2.443	Relapse
CIAT-98	Addiction	1.391	-1.158	-0.098	1.259	2.499	Salience
CIAT-99	Addiction	1.675	-0.579	0.721	1.796	2.730	Salience
CIAT-101	Dependent	1.504	-0.308	1.141	2.388	3.182	Relapse


*a, discrimination parameter; *b*, difficulty parameter.*

After the above steps, the final item bank of CAT-IA included 59 items with high discrimination, good item-fit, no DIF, and meeting the assumptions of IRT. The eighth column in Table 5 shows the domains of the 59 items: 6 items measured salience, 9 items measured tolerance, 6 items measured mood modification, 7 items measured relapse, 10 items measured withdrawal, 16 items measured negative outcomes, and 6 items measured benefits.

### Psychometric Properties of CAT-IA

**FIGURE 1:**
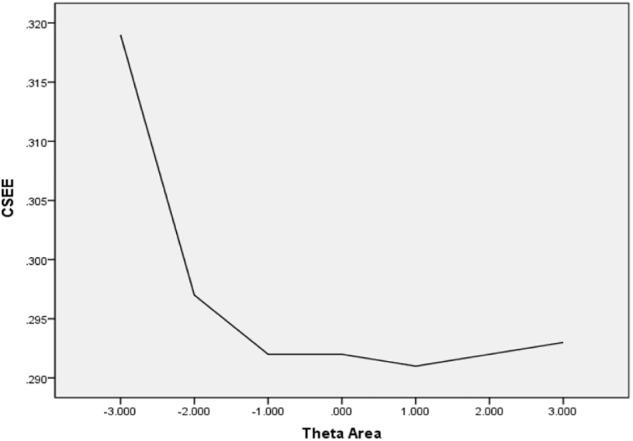
Conditional SEE (average SEE in each theta area).

**FIGURE 2:**
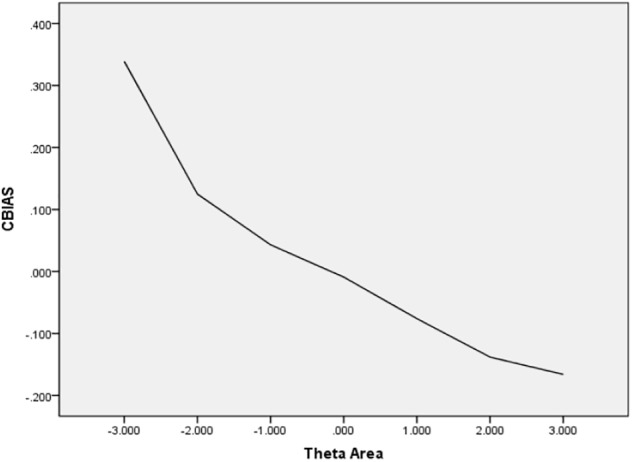
Conditional BIAS (average BIAS in each theta area).

**FIGURE 3:**
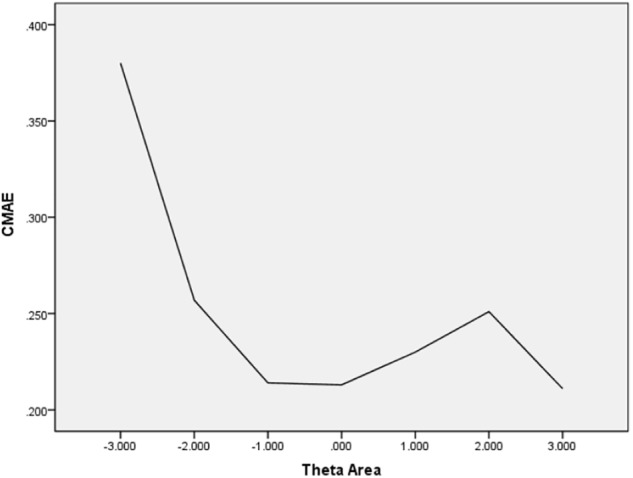
Conditional MAE (average MAE in each theta area).

**FIGURE 4:**
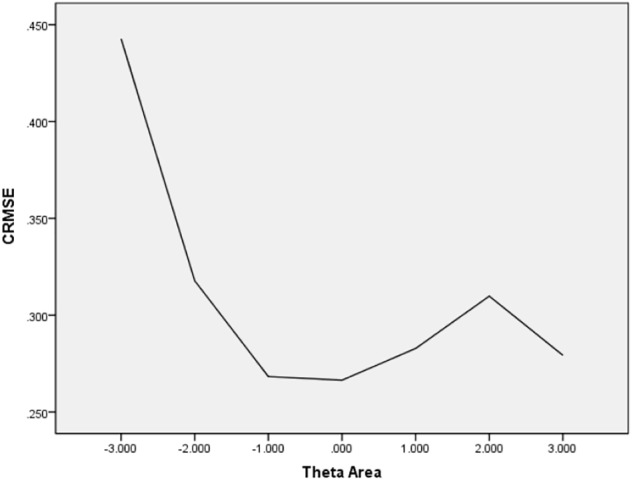
Conditional RMSE (average RMSE in each theta area).

In Table 6, the values of CBIAS, CMAE, CRMSE, and CSEE across all θ areas are displayed under several stopping rules. The second column documents the CSEE values across all θ areas, which ranged from 0.154 to 0.464. The values of CSEE across all θ areas that were less than the corresponding measurement precision decreased as measurement precision was made stricter. The third column reveals the values of CBIAS across all θ areas, which ranged from -0.016 to 0.008. Except for the stopping rule of SE (θ) ≤ 0.5, with CBIAS of -0.016 across all θ areas, the values of CBIAS across all θ areas decreased when the measurement precision was made stricter. The last two columns of Table 6 indicate that the CMAE and CRMSE values across all θ areas varied from 0.125 to 0.359, and 0.160 to 0.456, respectively. The values of CMAE and CRMSE across all θ areas decreased as measurement precision was made stricter, respectively. All these results indicated that the CAT-IA had high measurement accuracy in psychometric properties. The values of CBIAS, CMAE, CRMSE, and CSEE in each θ area under stopping rule SE (θ) ≤ 0.3 are displayed in Figure 1–4. Clearly, as shown in Figure 1, the CSEE values were closely commanded to less than 0.3 at -2 ≤ θ area. The values of CBIAS were inversely proportional to all θ areas. In addition, CBIAS values gradually decreased as the ability increased, as shown in Figure 2. The changing trends of CMAE and CRMSE were approximately consistent across all θ areas, as shown in Figure 3, 4. These results were consistent for all stopping rules.

**Table 6:** The psychometric properties of CAT-IA using CBIAS, CMAE, CRMSE, and CSEE indices across all θ areas.

Stopping rule	CSEE	CBIAS	CMAE	CRMSE
None	0.154	-0.005	0.125	0.160
SE (θ) ≤ 0.2	0.200	0.003	0.158	0.199
SE (θ) ≤ 0.3	0.292	0.007	0.227	0.283
SE (θ) ≤ 0.4	0.380	0.008	0.278	0.348
SE (θ) ≤ 0.5	0.464	-0.016	0.359	0.456


*None, all item bank was used; CBIAS, conditional bias; CMAE, conditional mean absolute error; CRMSE, conditional root mean square error; CSEE, conditional standard error of estimation.*

### Efficiency, Reliability, and Validity of CAT-IA

#### Efficiency and Reliability of CAT-IA

In Table 7, the CAT-IA simulation results are displayed under five measurement precision standards. As shown in the second column, the mean and SD of the items used both increased when the measurement precision was made stricter. In the third column, the mean SE of the latent traits for each stopping rule varied from 0.159 to 0.454. Except for the stopping rule of SE (θ) ≤ 0.2, the mean SEs were less than their corresponding measurement precision. Marginal reliability ranged from 0.794 to 0.973 with an average of 0.90, as shown in the fourth column. Evidently, marginal reliability increased as the measurement precision was made stricter. The last column in Table 7 shows the Pearson’s correlation between the estimated θ with stopping rule of None and the remaining stopping rules. The values of Pearson’s correlation ranged from 0.898 to 1 and were all significant at the 0.01 level (two-tailed), which showed that under different stopping rules, the algorithm of CAT-IA was effective. Table 7 also shows that the CAT-IA could greatly save item usage without loss of measurement precision. Under the stopping rule of SE (θ) ≤ 0.2, the Pearson’s correlation between the estimated theta by CAT-IA and the estimated theta by all of the items in the item bank reached 0.990; CAT-IA only used about half of the items (27.655 items) in the item bank. In brief, the CAT-IA saved 53.1% in item usage without loss of measurement precision. Under the two stopping rules of SE (θ) ≤ 0.3 and SE (θ) ≤ 0.4, the Pearson’s correlations were both above 0.90; CAT-IA thus saved 80.7 and 89.9% of item usage, respectively. All these results indicated that the CAT-IA had high efficiency and marginal reliability.

**Table 7:** CAT simulation statistics for CAT-IA under different stopping rules.

Stopping rule	Number of items used		Mean SE (theta)	Marginal reliability	*r*
				
	Mean	*SD*			
None	59	0	0.159	0.975	1
SE (θ) ≤ 0.2	27.655	12.070	0.203	0.959	0.990^**^
SE (θ) ≤ 0.3	11.380	9.064	0.293	0.914	0.962^**^
SE (θ) ≤ 0.4	5.952	4.819	0.380	0.856	0.932^**^
SE (θ) ≤ 0.5	3.675	2.000	0.454	0.794	0.898^**^


*None, all item bank was used; SD, standard deviation; SE, standard error; *r*, Pearson’s correlations. ^∗∗^ representing significant correlation at the 0.01 level (two-tailed).*

**FIGURE 5:**
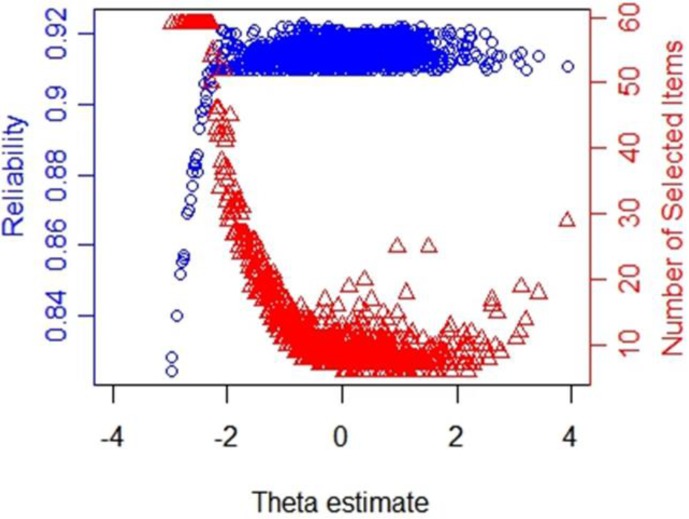
Number of selected items and reliability shown as a function of the final θ estimate under stopping rule SE (θ) ≤ 0.3.

The reliability and number of used items in CAT-IA on levels of the latent trait under stopping rule SE (θ) ≤ 0.3 are displayed in Figure 5. We noted a remarkable connection between the number of used items and reliability. Despite only using about 11.38 items, the CAT-IA obtained high reliability (above 0.9) and high measurement precision for a large number of individuals (estimated theta ranged from -2 to +4). Conversely, when the reliability was below 0.9, more items were used. This result was consistent for all stopping rules.

#### Concurrent Validity and Predictive Validity of CAT-IA

The Pearson’s correlations between the estimated θ of CAT-IA and the aggregate scores of IAT, GPIUS, GAS, and CIAT are documented in Table 8. The values of Pearson’s correlations varied from 0.646 to 0.944 and were all significant at the 0.01 level (two-tailed), which revealed that the CAT-IA had high concurrent validity. In addition, comparing the other scales, the correlation coefficient of CIAT was the highest under each stopping rule, whereas that of GAS was the lowest.

**Table 8:** Pearson’s correlations between the estimated θ of CAT-IA and the sum scores of four IA scales under different stopping rules.

Stopping rules	IAT	GPIUS	GAS	CIAT
None	0.862^∗∗^	0.861^∗∗^	0.754^∗∗^	0.944^∗∗^
SE (θ) ≤ 0.2	0.825^∗∗^	0.839^∗∗^	0.731^∗∗^	0.941^∗∗^
SE (θ) ≤ 0.3	0.781^∗∗^	0.796^∗∗^	0.684^∗∗^	0.926^∗∗^
SE (θ) ≤ 0.4	0.757^∗∗^	0.773^∗∗^	0.669^∗∗^	0.893^∗∗^
SE (θ) ≤ 0.5	0.728^∗∗^	0.740^∗∗^	0.646^∗∗^	0.858^∗∗^


*None, all item bank was used; IAT, Internet Addiction Test; GPIUS, Generalized Problematic Internet Use Scale; GAS, Gaming Addiction Scale; CIAT, Chinese Internet Addiction Test; ^∗∗^ representing significant correlation at the 0.01 level (two-tailed).*

The results of the predictive validity of CAT-IA are displayed in Table 9. All AUC values (with 95% confidence intervals) were above 0.71, indicating that CAT-IA had a large predictive effect (Rice and Harris, 2005). According to the large predictive effect, the cut-off point of IA was determined under each stopping rule for IAT and GAS, based on the values of sensitivity and specificity. For example, under the stopping rule of SE (θ) ≤ 0.2 in the diagnostic criteria of GAS, if the cut-off point of the 59-item bank was set to 0.801, the sensitivity and specificity of CAT-IA reached 0.922 and 0.862, respectively. These results showed that the CAT-IA had high predictive validity and had strong discrimination between individuals with IA disorder and healthy individuals.

**Table 9:** Area under the curve Statistics for the IAT and GAS scale under different stopping rules, and 95% confidence intervals.

Stopping rules	GAS				IAT			
		
	AUC [95% CI]	Cut-off	Se	Sp	AUC [95% CI]	Cut-off	Se	Sp
None	0.957 [0.933, 0.981]	0.750	0.969	0.838	0.931 [0.913, 0.950]	0.749	0.815	0.882
SE (θ) ≤ 0.2	0.948 [0.921, 0.976]	0.801	0.922	0.862	0.903 [0.887, 0.918]	0.203	0.865	0.773
SE (θ) ≤ 0.3	0.927 [0.895, 0.958]	0.946	0.813	0.893	0.875 [0.856, 0.893]	0.205	0.825	0.746
SE (θ) ≤ 0.4	0.919 [0.884, 0.954]	0.868	0.828	0.873	0.863 [0.844, 0.882]	0.151	0.835	0.728
SE (θ) ≤ 0.5	0.906 [0.868, 0.944]	0.780	0.797	0.860	0.848 [0.828, 0.868]	0.088	0.861	0.673


*None, all item bank was used; IAT, Internet Addiction Test; GAS, Gaming Addiction Scale. Se, sensitivity; Sp, specificity.*

## Discussion

CAT studies have focused on depression or anxiety for clinical individuals in the field of mental health (e.g., Fliege et al., 2005; Flens et al., 2016, 2017). However, to the best of our knowledge, there are no CAT studies on IA. In this research, we developed a CAT-IA to provide a new and effective assessment of IA. The original item bank of IA was subjected to psychometric evaluation; items were excluded until all of the remaining items in the item bank satisfied the requirements of psychometric evaluation. Subsequently, the efficiency, reliability, and validity of the final item bank of the CAT-IA were assessed under different stopping rules. The results showed that the final 59-item CAT-IA item bank met the three IRT assumptions, and possessed high discrimination, good item-model fit, and no DIF. Moreover, the CAT-IA could significantly save testing items and effectively reduce the test burden of participants, while also having high reliability, concurrent validity, and predictive validity.

Kocalevent et al. (2009) demonstrated that simulation and actual results of CAT tend to show high similarity. There are three reasons to implement actual CAT studies under different stopping rules. First, the same participants are used not only to estimate item parameters but also to simulate CAT studies, which could result in overfitting and more optimistic results (Friedman et al., 2010). Second, margin reliability and predictive validity might be overestimated because the data of CAT simulation studies come from the original database. Third, De Beurs et al. (2012) indicated that the results of a test are affected by the measurement tools. The original CAT study was done on a computer, but now it is conducted as a paper-and-pencil survey, which may lead to different outcomes.

When applying CAT-IA in clinical practice or research, CAT-IA may have different reliability results for different observers; that is, individuals of different abilities are provided with different information. For example, in the present study, under the stopping rule SE (θ) ≤ 0.3, reliability was very low and a large number of items were used when the individual has overly high or low abilities, indicating that small differences between two participants with either very high or very low abilities may not be detected, which was similar to Reise and Waller (2009) findings. To prevent the emergence of test bias, the reliability provided by the CAT-IA was set as similar and high for all test-takers. Nonetheless, we recognized the impact of the difficulty parameter distribution under the GRM. For example, in this study, there were no items to match persons whose abilities are below -1.968 in that the minimum value of the difficulty parameters was b1 = -1.968. Therefore, the CAT-IA provided these people with scarce information, and the measurement accuracy and reliability for them were very low despite the use of a large number of items of the 59-item bank. In future studies, researchers can increase the number of items with high or low difficulty parameter to make the difficulty parameter reasonable, which could not only provide high measurement accuracy and reliability for each participant but also greatly reduce the number of selected items for each person.

The standard IRT model is generally based on assumptions of unidimensionality and local independence. However, the single-dimensional and locally independent assumptions in real life may not be completely satisfied. For example, many researchers believe that the factor structure of IA should be multidimensional rather than unidimensional (e.g., Thatcher and Goolam, 2005; Lemmens et al., 2009; Caplan, 2010). Based on local dependency, Wainer et al. (2000a) proposed a widely used 3PL testlet model, in which dependent items did not need to be excluded when the testlet model was used in a CAT. According to these results, future studies can extend the unidimensional CAT into the multidimensional CAT and use the testlet model to solve local dependency between items.

In addition, concurrent validity in the present study was evaluated by Pearson’s correlations between the estimated θ. of CAT-IA and the aggregate scores of each scale. This method can result in item overlap that may overestimate the concurrent validity. Future studies should utilize other external scales to investigate concurrent validity. Further, De Beurs et al. (2012) proved that the same test applied in different situations may lead to changes in the measurement characteristics. Therefore, factorial invariance should be considered in future research. Lastly, although there are many methods for the selection of initial items, with respect to the estimation of latent trait, item selection, and exposure rate, this study failed to address enough methods (such as different parameter estimation and item selection methods), which should be fully considered in future studies.

## Ethics Statement

All procedures performed in studies involving human participants were in accordance with the ethical standards of the institutional and/or national research committee and with the 1964 Helsinki Declaration and its later amendments or comparable ethical standards. Informed consent was obtained from all individual participants included in the study. The current study was conducted in conformity to the recommendations of psychometrics studies on mental health at the Research Center of Mental Health, Jiangxi Normal University and approved by the Research Center of Mental Health, Jiangxi Normal University and the Ethics Committee of Psychology Department in Jiangxi Normal University. The written informed consent was obtained from all participants in accordance with the Declaration of Helsinki. All participants gave their written informed consent. The parental consent was also obtained for all participants under the age of 16.

## Author Contributions

YZ wrote the manuscript. YC and DT guided the manuscript writing and data processing. DW and XG processed the data.

## Conflict of Interest Statement

The authors declare that the research was conducted in the absence of any commercial or financial relationships that could be construed as a potential conflict of interest.
